# Effects of heat-killed *Enterococcus faecalis* T-110 supplementation on gut immunity, gut flora, and intestinal infection in naturally aged hamsters

**DOI:** 10.1371/journal.pone.0240773

**Published:** 2020-12-30

**Authors:** Takio Inatomi, Konosuke Otomaru

**Affiliations:** 1 Inatomi Animal Hospital, Tokyo, Japan; 2 Joint Faculty of Veterinary Medicine, Kagoshima University, Korimoto, Kagoshima, Japan; USDA-Agricultural Research Service, UNITED STATES

## Abstract

Infectious diseases are a threat to elderly individuals, whose immune systems weaken with age. Among the various infectious diseases, *Clostridium difficile* infection is associated with a high rate of mortality in elderly individuals and is a serious health problem worldwide, owing to the increasing infection rates. Probiotic use has been proposed as an effective countermeasure for *C*. *difficile* infection. The aim of this study was to evaluate the effects of heat-killed *Enterococcus faecalis* T-110 on intestinal immunity, intestinal flora, and intestinal infections, especially *C*. *difficile* infections, in naturally ageing animals, for extrapolating the results to elderly human subjects. Twenty female hamsters were randomly distributed into two groups. Group 1 was fed a basal diet and group 2 was fed a basal diet supplemented with heat-killed *E*. *faecalis* for 7 days. Heat-killed *E*. *faecalis* T-110 improved the gut immunity and microflora, especially *Clostridium perfringens* and *C*. *difficile*, in naturally aged hamsters. Therefore, heat-killed *E*. *faecalis* T-110 use may be a countermeasure against age-related immune dysfunction and intestinal infections, especially *C*. *difficile* infection, in elderly humans. However, further investigation in this regard is needed in humans.

## Introduction

Infectious diseases are a leading cause of mortality and significant morbidity in elderly individuals, who are at a greater risk than younger individuals [1]. With age, the humoral immunity and cell-mediated immunity are weakened against newly encountered pathogens or vaccines [2–6], necessitating countermeasures for age-related immune dysfunction.

Among the various infectious diseases, *Clostridium difficile* infection is a social problem in elderly individuals. *Clostridium difficile* produces a toxin that results in symptoms ranging from mild diarrhoea to inflammation of the bowel (pseudomembranous colitis), which can cause death. *Clostridium difficile*-associated diarrhoea is a severe form of diarrhoea in humans. There are three key risk factors associated with the development of this infection: antibiotic use [7], ageing [8], and hospitalisation [9]. *Clostridium difficile*-associated diarrhoea is responsible for approximately 10%–20% of all cases of antibiotic-associated diarrhoea [10], and it can occur up to 8 weeks after antibiotic therapy [11]. With the increasing threat of *C*. *difficile* infection, probiotics have been proposed as one of the effective countermeasures for *C*. *difficile* infection [12–14].

Probiotics have been defined as live, microbial, food components that are beneficial for human health. Recently, they have been shown to exhibit beneficial effects similar to those of live microbes; genetically engineered microbes and nonviable microbes are regarded as probiotics [15, 16]. Lactic acid bacteria, one of the most common types of probiotic bacteria, have been reported to exhibit beneficial effects on host homeostasis, including the activation of immune functions [17, 18]. To date, several heat-killed lactic acid bacteria have been shown to modulate specific and/or non-specific immune responses in animal models and occasionally in human subjects [19].

Regarding the use of probiotics for infection control, it is important that the probiotics administered are not infectious. *Enterococcus faecalis* T-110 (TOA Pharmaceutical Co., Ltd., Tokyo, Japan), which belongs to the group of lactic acid bacteria, is unlikely to be a causative agent of opportunistic infection [20]. *Enterococcus faecalis* T-110 is approved for medical use and is widely used in Japan, China, and India for the treatment and prevention of gastrointestinal infections such as *Salmonella* infection and rotavirus gastroenteritis. In terms of safety, *E*. *faecalis* T-110 is considered suitable for the treatment and prevention of gastrointestinal infections.

Several studies have shown that ageing affects the gut flora [21–24]. Generally, senescence-accelerated animals are often used to investigate the effects of ageing. However, a few studies have shown that the gut flora of senescence-accelerated animals is similar to that of naturally ageing animals. Stephan et al. [25] reported that the gut microbiota of laboratory mice differs from that of free-living mammals and humans, making them unsuitable for the study of gut immunity. *Clostridium difficile* is a bacterium endemic to the intestine of hamsters. Aged hamsters often suffer from diarrhoeal infections, especially *C*. *difficile* infections [26]. These factors suggest that they are the best model of *C*. *difficile* infections in aged animals. Challenge tests are generally conducted in bacterial infection tests, but they are considered unsuitable as infection models for indigenous intestinal bacteria caused by immunosuppression due to ageing. Only a few studies have investigated the effects of safety-guaranteed, heat-killed bacteria on intestinal immunity, gut flora, or intestinal infections in naturally aged animals. Therefore, the aim of this study was to evaluate the effects of heat-killed *E*. *faecalis* T-110 on intestinal immunity, flora, and infections in naturally ageing animals, for prospective extrapolation of such information to studies on elderly humans.

## Materials and methods

### Ethical approval

This study was conducted at Inatomi Animal Clinic in Tokyo Prefecture, Japan. It complied with the fundamental guidelines for the proper conduct of animal experiments and related activities in academic research institutions under the jurisdiction of the Ministry of Education, Culture, Sports, Science and Technology. It was approved by the Ethics Committee of the Inatomi Animal Clinic (Tokyo, Japan; approval number 2020–001).

### Animals, diets, and management

Twenty healthy, 547-day-old female hamsters (*Phodopus sungorus*) were purchased from Japan SLC, Inc., Hamamatsu, Japan, and acclimatised for 10 days before use in the experiments. These animals were healthy and did not receive any treatments before the study. They were randomly divided into two treatment groups (groups 1 and 2) of 10 hamsters each and housed individually in a cage (27 × 15 × 10 cm) under a 24 h light/dark cycle for 14 days. Temperature was maintained at 26 ± 1°C, and a basic diet (Rodent Diet CE-2, CLEA JAPAN, Tokyo, Japan) and water were provided to the hamsters *ad libitum*.

Group 2 hamsters received 0.1 ml of heat-killed *E*. *faecalis* T-110 saline suspension (1.0 × 10⁷ cfu/ml) daily from days 1 to 7. Heat-killed *E*. *faecalis* T-110, a commercial heat-killed and dried cell preparation (TOA Biopharma, Tokyo, Japan), was used. The heat-killed *E*. *faecalis* T-110 saline suspension was prepared as previously described [27]. The faeces of individual hamsters were checked daily during this experiment and was categorised according to a faecal score (0, normal faeces; 1, loose stool; 2, moderate diarrhoea; 3, severe diarrhoea).

After this experiment, all hamsters used in this study were bred under a normal environment without being euthanised.

### Immunological study

On days 1, 7, and 14, the total immunoglobulin A (IgA) concentration in the faeces of all hamsters was measured using a commercial enzyme-linked immunosorbent assay (ELISA) kit (Hamster Immunoglobulin A ELISA Kit; My BioSource, Inc, California, USA). The ELISA procedure was conducted according to the protocol of the manufacturer.

### Microbiological study

The faeces of hamsters were used for the microbiological study. Bacterial genomic DNA from the samples was extracted using a commercial extraction system (QuickGene 810 and Quick Gene DNA tissue kit; KURABO, Osaka, Japan), as previously described [28]. Quantitative real-time polymerase chain reaction (PCR) analyses of *Bifidobacterium* sp., *C*. *perfringens*, *Lactobacillus* spp., and *C*. *difficile* were performed using the Rotor-Gene system 6200 (Qiagen, Tokyo, Japan), as previously described [29]. The primer sequences and PCR conditions for each bacterium are given in Table 1.

**Table 1:** Primers and thermal cycling profiles used in this study.

Primer	Sequence	Annealing temperature (°C)	Reference
*Bifidobacterium* sp.-F	5′-GATTCTGGCTCAGGATGAACGC-3′	60°C	Gueimonde et al. [50]
*Bifidobacterium* sp.-R	5′-CTGATAGGACGCGACCCCAT-3′	60°C	Gueimonde et al. [50]
*Lactobacillus* group-F	5′-AGCAGTAGGGAATCTTCCA-3′	58°C	Rinttila et al. [51]
*Lactobacillus* group-R	5′-CACCGCTACACATGGAG-3′	58°C	Rinttila et al. [51]
*Clostridium perfringens*-F	5′-CGCATAACGTTGAAAGATGG-3′	60°C	Wise & Siragusa [52]
*Clostridium perfringens*-R	5′-CCTTGGTAGGCCGTTACCC-3′	60°C	Wise & Siragusa [52]
*Clostridium difficile*-F	5′-TTGAGCGATTTACTTCGGTAAAGA-3′	58°C	Rinttila et al. [51]
*Clostridium difficile*-R	5′-CCATCCTGTACTGGCTCACCT-3′	58°C	Rinttila et al. [51]

F–forward primer, R–reverse primer.

*Bifidobacterium* sp., *C*. *perfringens*, *Lactobacillus* spp., and *C*. *difficile* in the faeces were quantified by real-time PCR, with three replicates for each sample. Amplification was carried out with a 10-μl reaction mixture containing 5 μl of SYBR_Premix Ex Taq (Takara Bio, Shiga, Japan), 1 μl of DNA template, and 0.2 μmol/l of each primer. Primers for *Bifidobacterium* sp., *C*. *perfringens*, *Lactobacillus* spp., and *C*. *difficile* and the thermal programs are listed in Table 1. For each reaction described above, the positive control and negative water control were assayed together with samples. The melting curves of the amplified DNA were generated to verify the specificity of the reaction. To construct standard curves, 10-fold diluted target species genomic DNA preparations (between 0.1 pg and 10 ng; approximately 30–100 to 3.0 × 10⁶–1.0 × 10⁷ target genomes) were applied by PCR. A mixture of DNA isolated from an extensive set of non-target test bacteria (100 pg each) was used as the negative control. Standard curves of individual real-time PCR assays were used to quantify the target bacterial DNA from faecal DNA preparations. For each assay, the PCR results were converted to the average estimate of target bacterial genomes present in 1 g of faeces (wet weight).

### Statistical analysis

Values are presented as mean ± standard error. Mann–Whitney U-test was applied to analyse differences between mean values in all parameters. Differences between mean values were considered significant at *P* < 0.05 in all statistical analyses. Mann–Whitney U-test was performed using EZR software (Saitama Medical Center, Jichi Medical University); EZR is a graphical user interface for R (The R Foundation for Statistical Computing, version 2.13.0) [30]. The significance level was set at *P* < 0.05.

## Results

### Total number of days of abnormal defaecation

In the first week, the total number of days of abnormal defaecation improved in group 2 compared with that in group 1 (*P* < 0.05) (Table 2). In the second week, there was no difference in the total number of days of abnormal defaecation in groups 1 and 2.

**Table 2:** Total number of days apparent abnormal defaecation in hamsters fed a basal diet (group 1) and a 1.0 × 10⁷ cfu/ml supplement of heat-killed *Enterococcus faecalis* (group 2).

	Days 1–7	Days 8–14
Group 1	1.4 ± 0.3ª	1.2 ± 0.2
Group 2	0.3 ± 0.2^b^	0.9 ± 0.2

^a, b^ Different letters within columns indicate differences between the treatment groups (*P* < 0.05).

### Immunological study

The total immunoglobulin A (IgA) concentration in faeces was significantly higher in group 2 than that in group 1 on day 7 (Table 3). On days 1 and 14, no difference in total immunoglobulin A (IgA) concentration was detected between groups 1 and 2.

**Table 3:** Total immunoglobulin A (IgA) concentration (mg/g) in the faeces of hamsters fed a basal diet (group 1) and a 1.0 × 10⁷ cfu/ml supplement of heat-killed *Enterococcus faecalis* (group 2).

	Day 1	Day 7	Day 14
Group 1	2.12 ± 0.09	1.98 ± 0.08^a^	2.01 ± 0.12
Group 2	2.08 ± 0.09	2.31 ± 0.08^b^	1.99 ± 0.11

^a, b^ Different letters within columns indicate differences between the treatment groups (*P* < 0.05).

### Microbiological study

On day 1, the counts of *Bifidobacterium* sp., *C*. *perfringens*, *Lactobacillus* spp., and *C*. *difficile* in faeces were not significantly different between the two groups (Table 4). On day 7, the counts of *C*. *perfringens* and *C*. *difficile* in the faeces were lower in group 2 than in group 2. The counts of *Bifidobacterium* sp. and *Lactobacillus* spp. were not significantly different between the two groups. After day 14, the counts of *Bifidobacterium* sp., *C*. *perfringens*, *Lactobacillus* spp., and *C*. *difficile* were similar in the faeces between the two groups.

**Table 4:** Microbiological analyses of faeces (log cells/g) of hamsters fed a basal diet (group 1) and a 1.0 × 10⁷ cfu/ml supplement of heat-killed *Enterococcus faecalis* (group 2).

	Day	Group 1	Group 2
*Lactobacillus* spp.	1	5.72 ± 0.20	5.62 ± 0.20
	7	5.40 ± 0.16	5.45 ± 0.20
	14	5.62 ± 0.24	5.85 ± 0.23
*Bifidobacterium* sp.	1	5.35 ± 0.12	5.53 ± 0.21
	7	5.60 ± 0.14	5.82 ± 0.22
	14	5.61 ± 0.14	5.88 ± 0.15
*Clostridium perfringens*	1	5.83 ± 0.07	5.69 ± 0.11
	7	5.72 ± 0.13^a^	4.51 ± 0.13^b^
	14	5.47 ± 0.04	5.40 ± 0.09
*Clostridium difficile*	1	5.34 ± 0.05	5.14 ± 0.10
	7	5.48 ± 0.06^a^	4.25 ± 0.09^b^
	14	5.62 ± 0.06	5.67 ± 0.06

^a, b^ Different letters within rows indicate differences between the treatment groups (*P* < 0.05).

## Discussion

Immunoglobulin A is one of the main defence elements that prevent pathogenic microorganisms from crossing the intestinal epithelial cell barrier and is important in protecting the intestinal mucosa [31, 32]. In the present study, heat-killed *E*. *faecalis* T-110 increased total immunoglobulin A (IgA) concentration in the faeces (Table 3). Similar effects have been reported by other studies [33–38]. Havenaar and Spanhaak [39] demonstrated that probiotics stimulate the immunity of animals in two ways: i) the probiotic flora migrates throughout the gut wall and multiplies to a limited extent and ii) antigens released by dead microorganisms are absorbed and stimulate the immune system. Kaji et al. [40] demonstrated that bacterial cell components of heat-killed probiotics stimulate the immune system. Their detailed mechanisms of action are unknown and further investigation into the immunostimulatory effect of cell wall components is needed. However, it has been suggested that heat-killed *E*. *faecalis* T-110 stimulates gut immunity and increases the production of IgA, consistent with the results of previous studies.

In this study, heat-killed *E*. *faecalis* T-110 decreased the number of *C*. *perfringens* and *C*. *difficile* in aged hamsters (Table 4). Similar effects have been reported in other studies [41–48]. Considering the increased faecal IgA concentration in the current study, it is likely that heat-killed *E*. *faecalis* T-110 decreased the number of *C*. *perfringens* and *C*. *difficile* by improving the gut immunity of ageing animals, consistent with the results of previous studies. Currently, drug resistance and adverse effects are a problem in the antimicrobial treatment of *C*. *difficile* infection. The results of the present study suggest that the use of antimicrobials in combination with heat-killed *Enterococcus faecalis* T-110 for *C*. *difficile* infection can reduce the amount of antimicrobials used. This may overcome the problem of drug resistance and adverse effects associated with antimicrobial treatment.

In this study, heat-killed *E*. *faecalis* T-110 decreased the total number of days of abnormal defaecation. *Clostridium perfringens* and *C*. *difficile* cause diarrhoea in hamsters [49]. Considering the decreased number of *C*. *perfringens* and *C*. *difficile* in the current study, it is likely that heat-killed *E*. *faecalis* T-110 decreased the total number of days of abnormal defaecation by improving the gut immunity in ageing animals.

## Conclusions

The administration of heat-killed *E*. *faecalis* T-110 decreased the counts of *C*. *perfringens* and *C*. *difficile* in aged hamsters. The suppression of *C*. *perfringens* and *C*. *difficile* by heat-killed *E*. *faecalis* T-110 administration could be partially associated with intestinal immunostimulation. Further investigations, including the identification of immunostimulatory cell wall components, are needed. However, based on the results of the present study, it can be concluded that heat-killed *E*. *faecalis* T-110 has the potential to improve intestinal environment, particularly in aged animals with *C*. *difficile* infection.
